# Supplementary material for: Evaluation of Gut Microbiota Stability and Flexibility as a Response to Seasonal Variation in the Wild François’ Langurs (Trachypithecus francoisi) in Limestone Forest
Source: Microbiol Spectr. 2023 Jul 5;11(4):e05091-22. doi: 10.1128/spectrum.05091-22 (PMC10433995; doi:10.1128/spectrum.05091-22)
Supplement: Supplemental file 2 — Supplemental material. Download spectrum.05091-22-s0002.pdf, PDF file, 1.9 MB [file spectrum.05091-22-s0002.pdf]

Evaluation of gut microbiota stability and flexibility as a response to seasonal variation in the wild François' langurs (*Trachypithecus francoisi*) in limestone forest

Hongying Liu[1,2,3], Yuhui Li[1,2,3], Jipeng Liang[4], Dengpan Nong[4], Youbang Li[1,2,3], Zhonghao Huang[1,2,3]*

[1]Key Laboratory of Ecology of Rare and Endangered Species and Environmental Protection (Guangxi Normal University), Ministry of Education, Guilin, China

[2]Guangxi Key Laboratory of Rare and Endangered Animal Ecology, Guangxi Normal University, Guilin, China

[3]College of Life Sciences, Guangxi Normal University, Guilin, China

[4]Administration Center of Guangxi Chongzuo White-headed Langur National Nature Reserve, Chongzuo, China

*** Correspondence:**
Dr. Zhonghao Huang, Guangxi Normal University, No.1 Yanzhong Road, Guilin, Guangxi, China.
Email: hzh773@126.com

Supplementary Information: results

Table S1 Sampling information of 152 fecal samples on gut microbiota analysis

Time	site	season	coordinate position	size	number
2019-2	Nonghengluliu Mountain	dry season	107°24′46.48″E-22°35′22.08″N	9	C058-C066
2019-4	Nonghengjian Mountain	rainy season	107°24′30.07″E-22°35′19.63″N	15	C089-C103
2019-5	Nonghengluliu Mountain	rainy season	107°24′46.48″E-22°35′22.08″N	11	C104-C114
2019-6	Nonghengluliu Mountain	rainy season	107°24′46.48″E-22°35′22.08″N	15	C115-C129
2019-7	Nonghengluliu Mountain	rainy season	107°24′46.48″E-22°35′22.08″N	12	C144-C155
2019-8	Nonghengjian Mountain	rainy season	107°24′30.07″E-22°35′19.63″N	10	C156-C165
2019-11	Nonghengluliu Mountain	dry season	107°24′46.48″E-22°35′22.08″N	11	TH001-TH011
2019-12	Nongmaimada Mountain	dry season	107°23′59.81″E-22°34′53.49″N	12	TH012-TH023
2020-1	Nonghengluliu Mountain	dry season	107°24′30.07″E-22°35′19.63″N	12	TH024-TH035
2020-2	Nonghengjian Mountain	dry season	107°24′30.07″E-22°35′19.63″N	12	TH036-TH047
2020-3	Nonghengluliu Mountain	dry season	107°24′46.48″E-22°35′22.08″N	11	TH048-TH058
2020-4	Nonghengluliu Mountain	rainy season	107°24′46.48″E-22°35′22.08″N	10	TH059-TH068
2020-5	Nonghengjian Mountain	rainy season	107°24′30.07″E-22°35′19.63″N	12	TH069-TH080
**Tatal**				**152**	

Table S2 Sampling information of 137 fecal samples on hormone assays

Time	season	size	number
2020-7	rainy season	14	TH091-TH104a
2020-8	rainy season	15	TH104b-TH118
2020-11	dry season	14	TH156-TH169
2020-12	dry season	14	TH170-TH183
2021-1	dry season	11	TH184-TH194
2021-2	dry season	13	TH195-TH207
2021-3	dry season	14	TH208-TH221
2021-4	rainy season	15	TH222-TH236
2021-5	rainy season	13	TH237-TH249
2021-6	rainy season	14	TH250-TH263
**Tatal**		**137**	

Table S3 Materials and reagents for DNA extraction, PCR and sequencing

Materials and reagents	Manufacturers	Function
E.Z.N.A.® Soil DNA Kit	Omega Bio-Tek (America)	DNA extraction
NanoDrop2000	Thermo Fisher Scientific (America)	Check the purity of the DNA
FastPfu Polymerase	TransGen (China)	PCR amplification
biowest agArose	Biowest (Spain)	Quality detection of DNA and PCR products
DYY-6C	Beijing Liuyi Biotechnology (China)	Electrophoresis
GeneAmp® 9700	ABI (America)	PCR amplification
AxyPrep DNA Gel Extraction Kit	Axygen Biosciences (America)	Purification of PCR products
Quantus™ Fluorometer	Promega (America)	Quantification of PCR products
NEXTFLEX Rapid DNA-Seq Kit	Bioo Scientific (America)	Building library
MiSeq Reagent Kit v3<br>Illumina Miseq	Illumina (America)	Sequencing

Table S6 Statistics for seasonal difference in alpha diversity of gut microbiota (GLMM)

**Bacteria Taxa**	**Explanatory variable**	**Estimated values**	**Standard error**	***t* value**
Shannon	Intercept	0.611	0.011	54.012
	Rainy season	0.009	0.008	1.077
*inv*simpson	Intercept	1.338	0.061	21.866
	Rainy season	0.048	0.041	1.151
ACE	Intercept	2.816	0.019	148.282
	Rainy season	0.044	0.017	2.549
Chao	Intercept	2.826	0.018	160.451
	Rainy season	0.033	0.016	2.045

Table S7 Results for seasonal difference in alpha diversity of gut microbiota (GLMM)

**Alpha index**	**Rainy season**	**Dry season**	χ^2 **(df = 1)**	***P* (*P* < 0.05)**
Shannon	4.25 ± 0.39	4.06 ± 0.41	1.281	0.258
*inv*simpson	30.03 ± 16.05	23.02 ± 11.08	1.451	0.228
ACE	725.38 ± 192.89	668.59 ± 112.56	5.236	0.022
Chao	719.19 ± 171.52	679.06 ± 107.72	3.191	0.074

Table S8 VIF analysis results for climatic factors

		Mean temperature	Maximum temperature	Rainfall	Sunshine duration	Relative humidity	Minimum humidity
VIF	After removing	40.53	41.34	4.86	3.98	9.76	10.24
	Before removing	6.99	removing	2.67	3.94	3.58	2.68

Table S9 Effects of climatic factors on gut microbiota of François' langurs

Climatic factors	Correlation of coefficients between climatic factors and sorting axises		(R^2) Coefficient of determination	*p* value
**Phylum level**				
	Axis1	Axis2		
Mean temperature	-0.414	0.910	0.038	0.048
Rainfall	-0.490	-0.871	0.035	0.074
Sunshine duration	0.516	0.856	0.037	0.054
Relative humidity	-0.960	-0.279	0.068	**0.003****
Minimum humidity	-0.901	-0.433	0.136	**0.001****
**Family level**				
**Climatic factors**	**Correlation of coefficients between climatic factors and sorting axises**		**(R^2) Coefficient of determination**	***p* value**
	**Axis1**	**Axis2**		
Mean temperature	0.518	-0.855	0.241	**0.001****
Rainfall	-0.414	-0.910	0.138	**0.001****
Sunshine duration	0.945	-0.327	0.089	0.094
Relative humidity	0.044	-0.999	0.274	**0.001****
Minimum humidity	0.416	-0.910	0.466	**0.001****

"**" $p < 0.01$

Table S11 Statistics for seasonal difference in Thyroid hormones (GLMM)

Thyroid hormones	Explanatory variable	Estimated values	Standard error	*t* value
T3	Intercept	18.562	0.205	90.652
	Rainy season	0.471	0.260	1.812
T4	Intercept	33.710	0.396	85.142
	Rainy season	0.180	0.503	0.357

Table S12 Seasonal difference test in the concentration of thyroid hormones (GLMM)

Thyroid hormones	Rainy season	Dry season	χ^2 (df = 1)	*P* (*P* < 0.05)
T3	19.034 ± 1.460	18.617 ± 1.480	2.765	0.096
T4	33.945 ± 2.908	33.801 ± 2.773	0.089	0.766

Table S13 The bacteria taxa that have an important influence on the fluctuation of T3 and T4 (random forest model)

T3

**Bacteria Taxa**	**Phylum level**		**Bacteria Taxa**	**Family level**	
	**IncNodePurity**	**p value**		**IncNodePurity**	**p value**
Cyanobacteria	3332.501	0.139	Enterobacteriaceae	1936.248	**0.040***
Myxococcota	3331.247	0.109	Eubacterium Coprostanoligenes group	1660.155	0.139
Proteobacteria	3236.250	0.148	norank_o__Chloroplast	1561.134	0.109
Bdellovibrionota	3166.924	**0.049***	Micromonosporaceae	1448.286	0.178
			norank_o__Clostridia_ vadinBB60_group	1183.121	0.287
			norank_o__ Izemoplasmatales	887.838	0.545
			Planococcaceae	858.127	0.574
			Christensenellaceae	802.486	0.633
			Butyricicoccaceae	801.763	0.614
			Vicinamibacteraceae	759.774	0.713
			Mitochondria	694.789	0.792
			Burkholderiaceae	636.870	0.812

T4

**Bacteria Taxa**	**Phylum level**		**Bacteria Taxa**	**Family level**	
	**IncNodePurity**	**p value**		**IncNodePurity**	**p value**
Bacteroidetes	16610.49	0.198	Bacillaceae	8265.514	0.119
Chloroflexi	16523.53	0.168	unclassified_o__ Micrococcales	6980.868	0.356
unclassified_k__ norank_d__Bacteria	16142.97	0.257	Prevotellaceae	6885.299	0.278
Methylomirabilota	15669.77	0.188	norank_o__Peptostreptoco ccales.Tissierellales	6743.367	0.317
			norank_o__Veillonellales. Selenomonadales	6020.664	0.297
			Nocardioidaceae	5750.232	0.246
			Veillonellaceae	5667.100	0.287
			Beijerinckiaceae	5665.390	0.356
			Butyricicoccaceae	5357.924	0.356
			Akkermansiaceae	4744.668	0.584
			Ruminococcaceae	4439.196	0.673
			unclassified_c__ Actinobacteria	4233.784	0.703

"*" $p < 0.05$

Table S14 Statistics for seasonal difference in activity budget (GLMM)

**Activity**	**Explanatory variable**	**Estimated values**	**Standard error**	***t* value**
Resting	Intercept	0.662	0.027	24.330
	Rainy season	-0.004	0.038	-0.103
Moving	Intercept	0.258	0.019	13.338
	Rainy season	0.008	0.027	0.282
Feeding	Intercept	0.036	0.011	3.393
	Rainy season	0.010	0.015	0.644
Grooming	Intercept	0.011	0.002	4.823
	Rainy season	-0.002	0.003	-0.679
Playing	Intercept	0.032	0.005	6.233
	Rainy season	-0.011	0.007	-1.535

Table S15 Examination of seasonal differences in activity budget (GLMM)

Activity	Rainy season	Dry season	χ^2 (df = 1)	***P* (*P* < 0.05)**
Resting	65.79% ± 6.31%	66.19% ± 4.40%	0.013	0.908
Moving	26.60% ± 4.50%	25.83% ± 3.13%	0.099	0.753
Feeding	4.61% ± 2.80%	3.63% ± 1.16%	0.505	0.477
Grooming	0.90% ± 0.38%	1.12% ± 0.54%	0.560	0.454
Playing	2.11% ± 1.07%	3.23% ± 1.00%	2.583	0.108

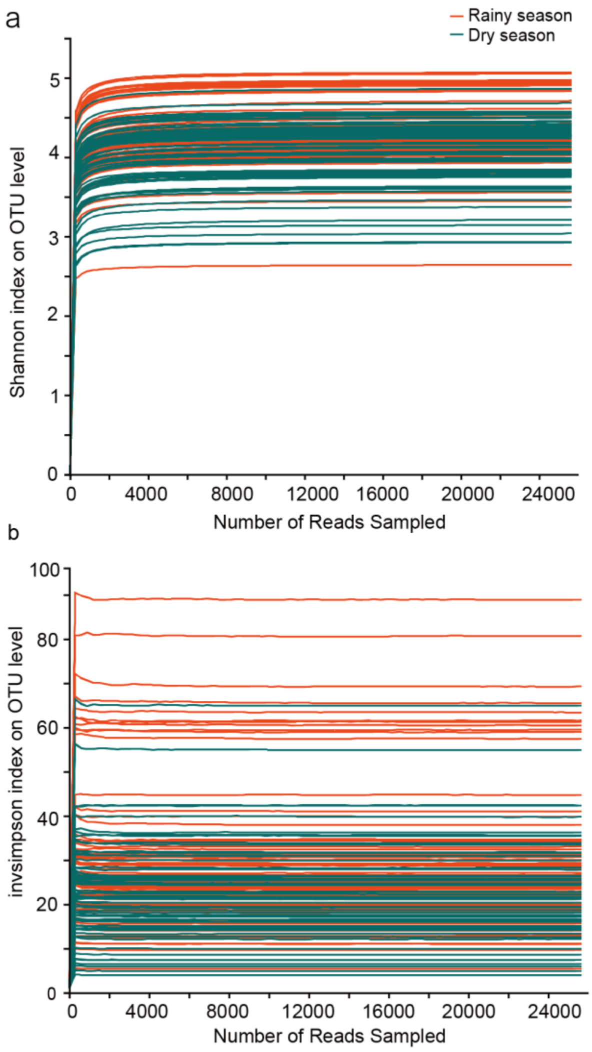

Fig. S1 Rarefaction curves show sufficient sequencing depth.

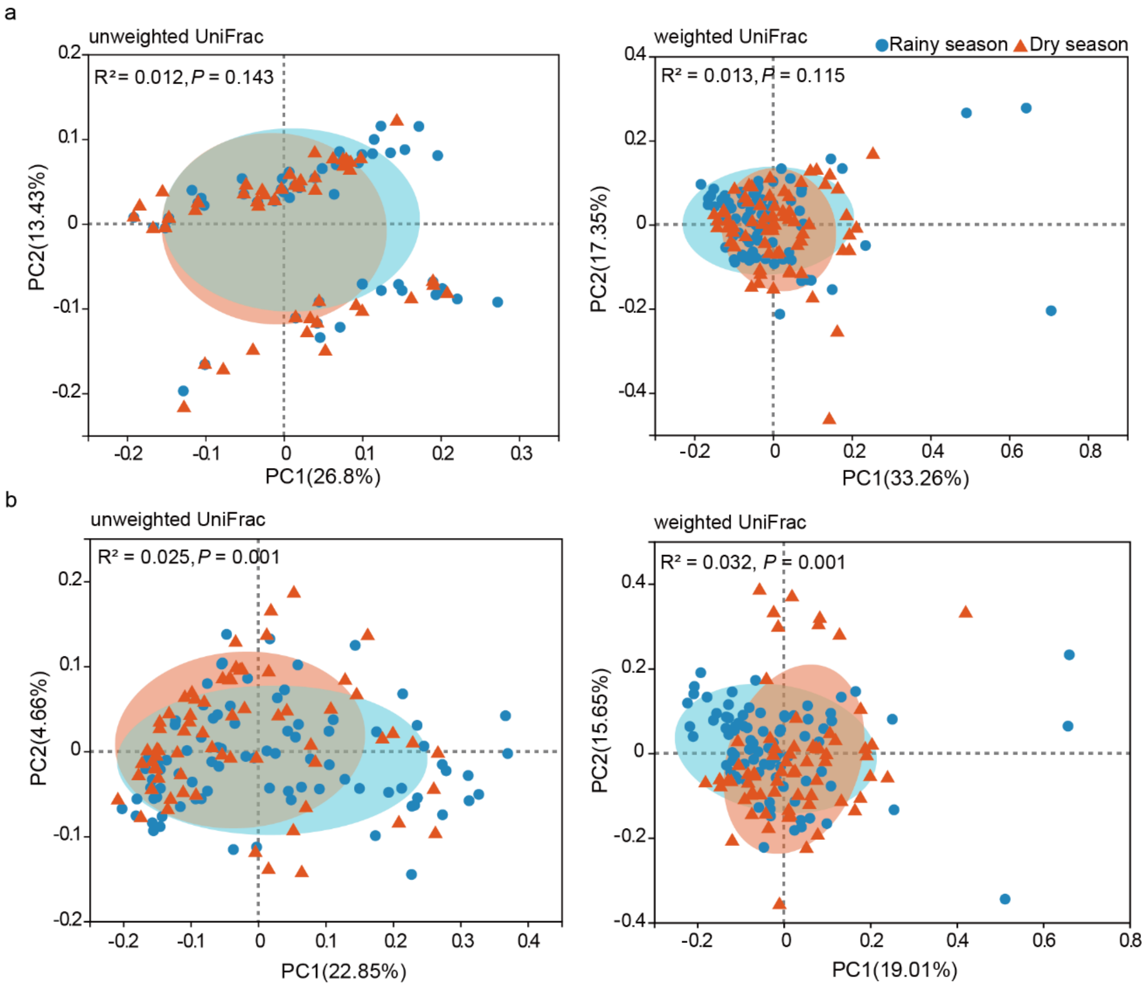

Fig. S2 Seasonal Comparison of gut microbiota Beta diversity at the phylum (a) and family level (b) (Tested by PERMANOVA, $P < 0.05$).

.

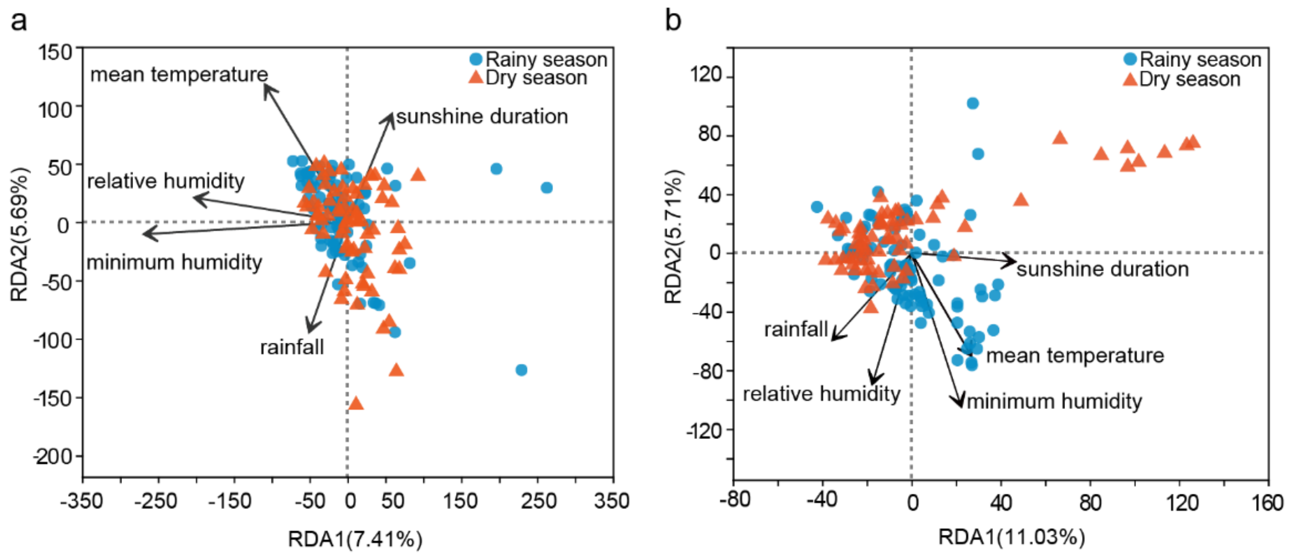

Fig. S3 Redundancy analysis (RDA) of climatic factors and gut microbiota of François' langurs (circles and triangles represent dry season and rainy season samples, respectively, Black arrows represent climate factors)

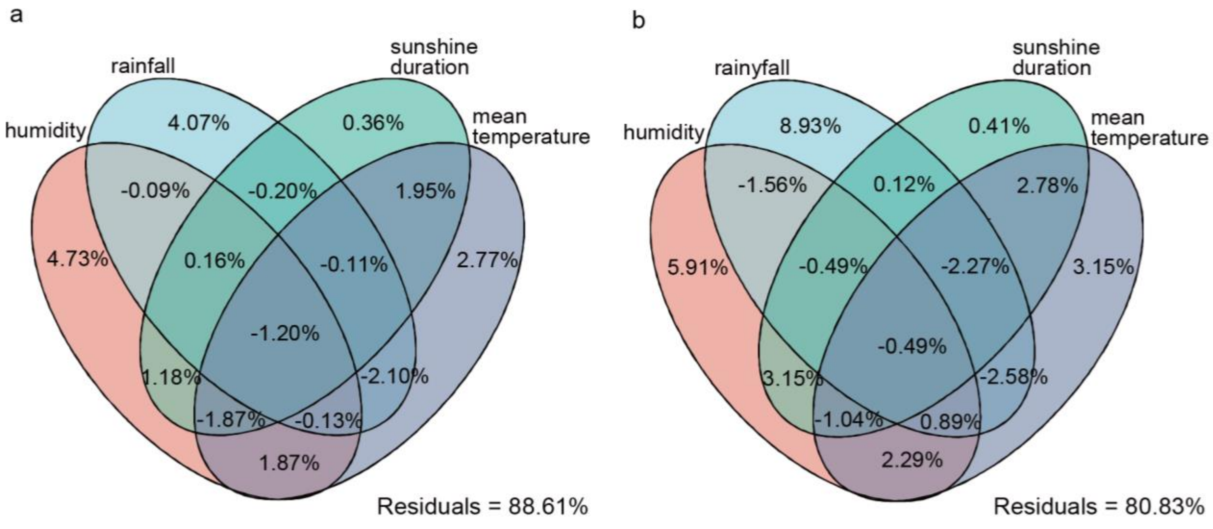

Fig. S4 The explanation power of climatic factors for seasonal differences in gut microbiota of François' langurs (Different colored ellipses represent different climatic factors; the intersecting part of the ellipse represent the common explanation, and the non-intersecting part represent the single explanation, and negative percentage represents variables explain less variation than random normal variables. "Residuals" represents the community differences that cannot be explained by the climatic factors)

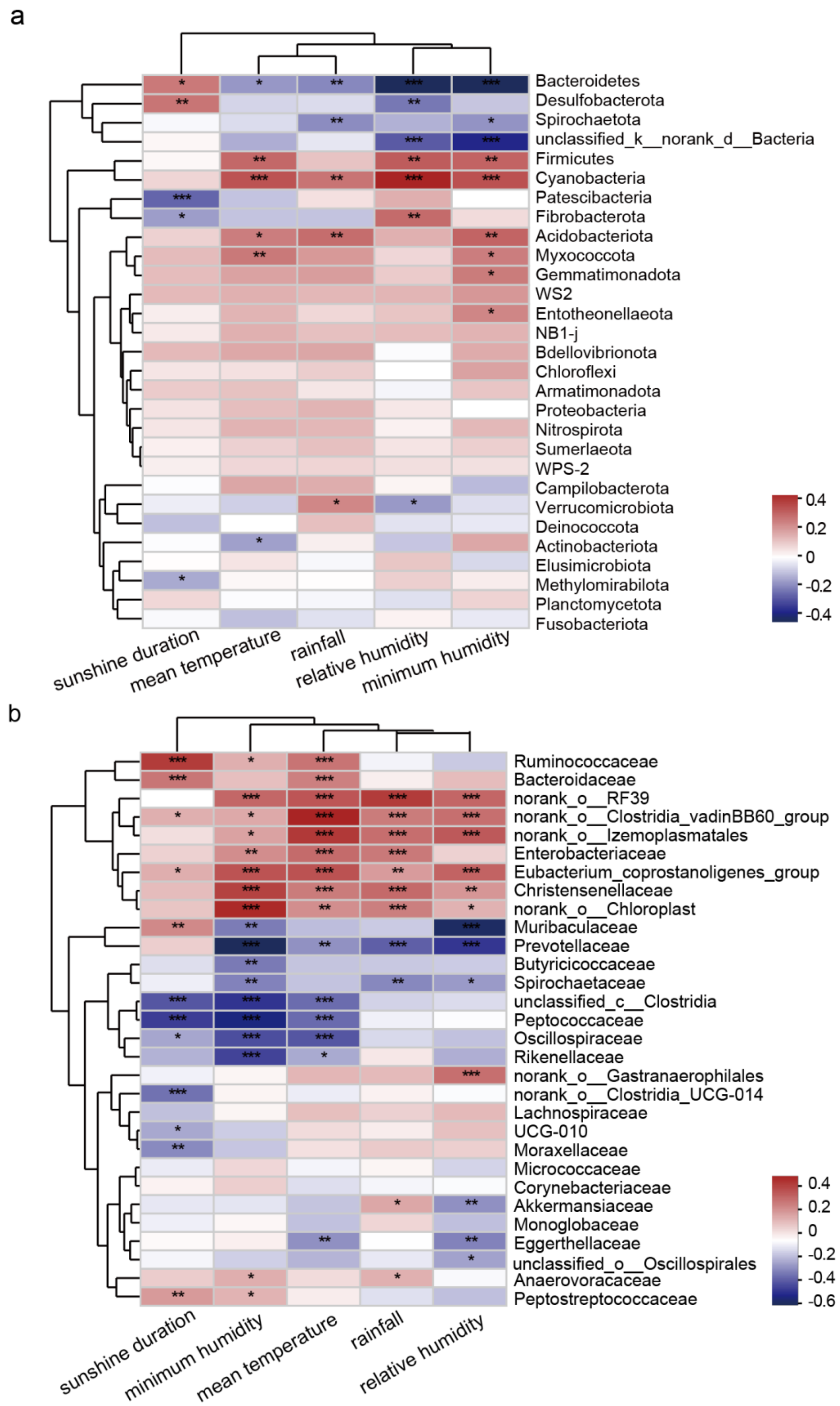

Fig. S5 Heat map showing correlations among climatic factors and gut microbiota of François' langurs (a: phylum level; b: family level (top 30), "*" means significant difference $p < 0.05$)

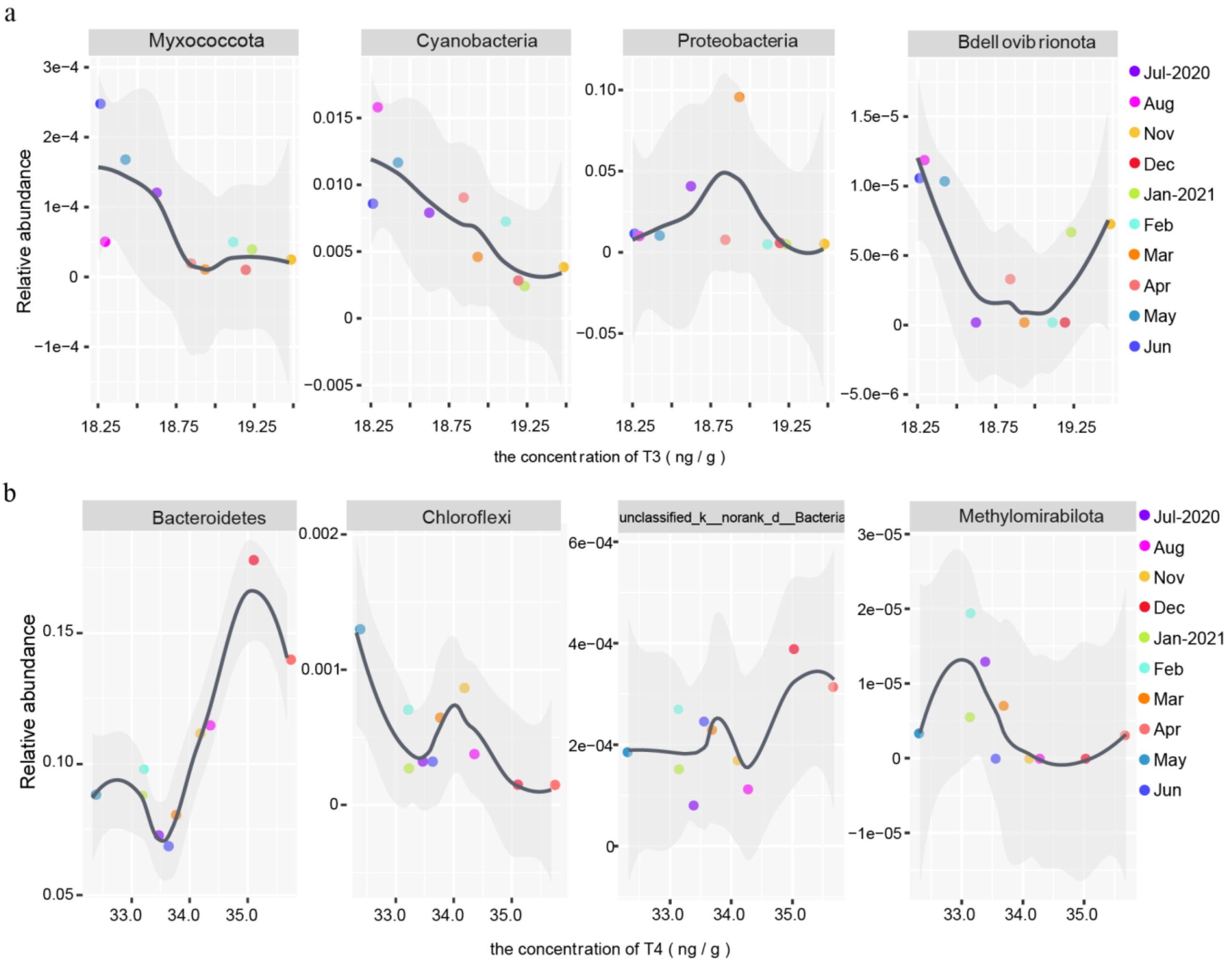

Fig. S6 Correlation between the abundance of important bacterial taxa at the phylum level of François' langurs and the fluctuation of T3 (a) and T4 (b) through the random forest model.

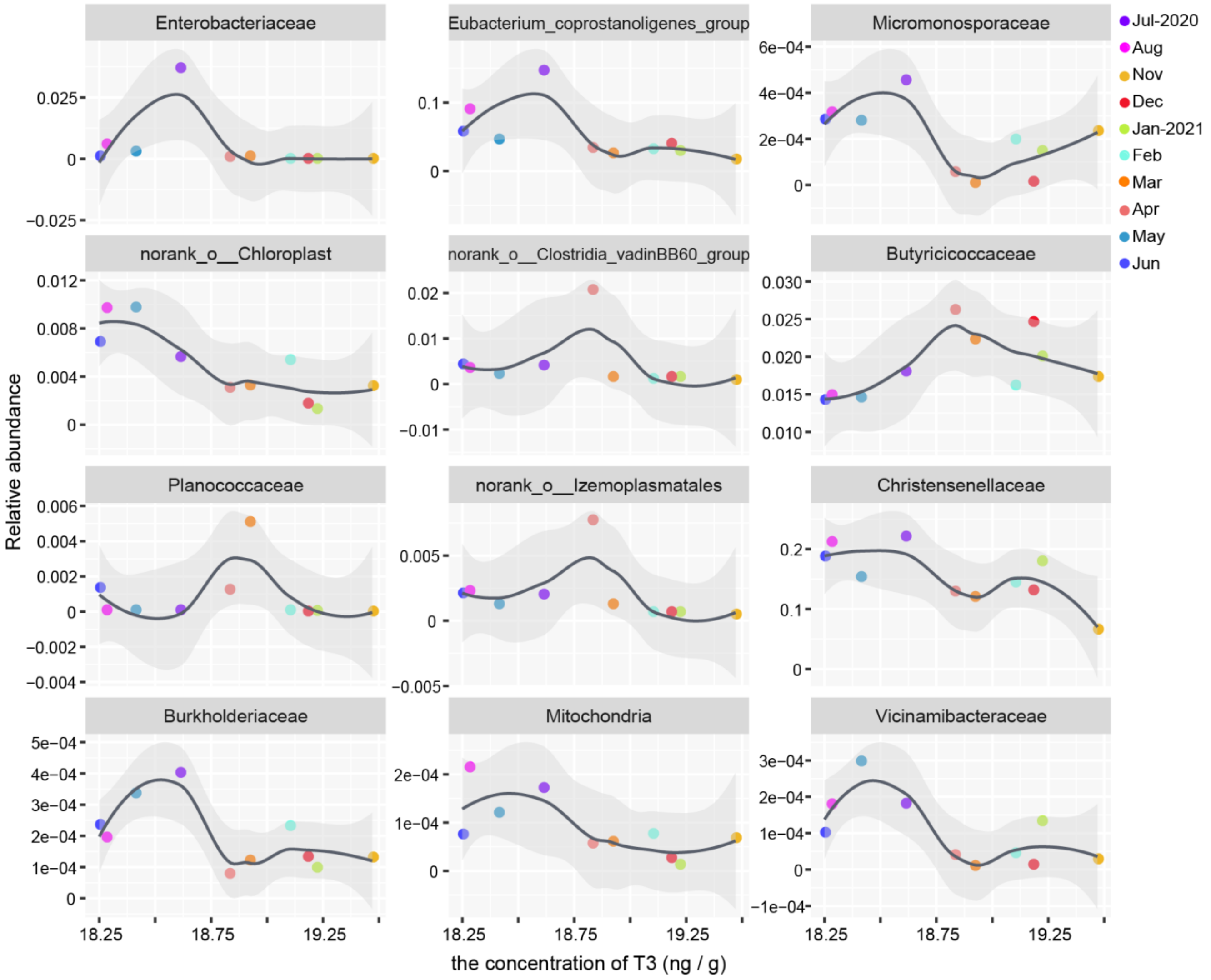

Fig. S7 Correlation between the abundance of important bacterial taxa at the family level of François' langurs and the fluctuation of T3 through the random forest model.

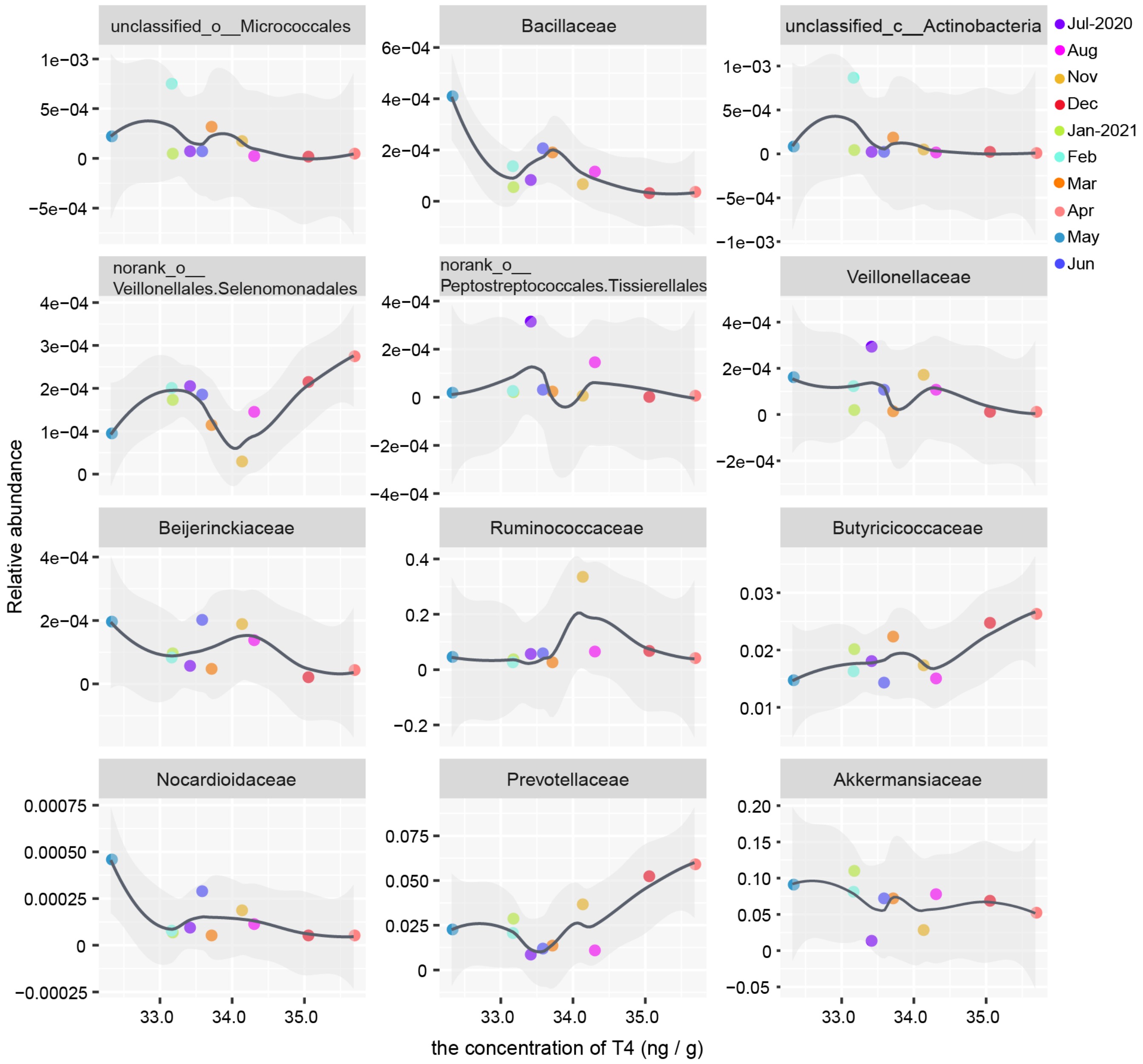

Fig. S8 Correlation between the abundance of important bacterial taxa at the family level of François' langurs and the fluctuation of T4 through the random forest model.
